# Three Carbazole-Based Polymers as Potential Anodically Coloring Materials for High-Contrast Electrochromic Devices

**DOI:** 10.3390/polym9070284

**Published:** 2017-07-18

**Authors:** Yuh-Shan Su, Tzi-Yi Wu

**Affiliations:** Department of Chemical and Materials Engineering, National Yunlin University of Science and Technology, Yunlin 64002, Taiwan; d10115003@yuntech.edu.tw

**Keywords:** electrochemical polymerization, optical contrast, spectroelectrochemistry, coloration efficiency, electrochromic devices

## Abstract

Three carbazole-based conjugated polymers (poly(3,6-di(2-thienyl)carbazole) (PDTC), poly(2,7-bis(carbazol-9-yl)-9,9-spirobifluorene) (PS2CBP), and poly(3,6-bis(*N*-carbazole)-*N*-ethylcarbazole) (PCEC)) are synthesized using electrochemical polymerization. The spectroelectrochemical studies indicate that the PDTC, PS2CBP, and PCEC films show reversible electrochromic behaviors in their redox states, and the PS2CBP film shows a distinct color transition with four various colors (gray at 0 V, grayish-green at 1.0 V, moss green at 1.2 V, and foliage green at 1.4 V). The maximum optical contrast of the PS2CBP and PCEC films is 39.83% at 428 nm and 32.41% at 420 nm, respectively, in an ionic liquid solution. Dual-type electrochromic devices (ECDs) that employ PDTC, PS2CBP, or PCEC film as an anodic layer, and PProDOT-Et_2_ film as a cathodic layer, were constructed. The as-prepared PCEC/PProDOT-Et_2_ ECD shows high optical contrast (38.25% at 586 nm) and high coloration efficiency (369.85 cm^2^ C^−1^ at 586 nm), and the PS2CBP/PProDOT-Et_2_ ECD shows high optical contrast (34.45% at 590 nm), good optical memory, and good long-term cycling stability.

## 1. Introduction

In electrochromism, it is widely known that an electrochromic material is able to change its color reversibly at different potentials [1]. Electrochromic material can be divided into two classes: inorganic and organic electrochromic materials. Common inorganic electrochromic materials include WO_3_ and V_2_O_5_ [2], whereas viologens and conjugated polymers are common organic electrochromic materials [3,4]. Electrochromic materials are widely used in smart windows/mirrors, displays [2], electrochromic devices (ECDs) [5,6], and electronic paper [7]. Among these applications, the use of organic electrochromic materials as cathodically coloring material in electrochromic devices has become popular due to their ease of chemical synthesis, high coloration efficiency, short switching time, high optical contrast, low oxidation potential, and good long-term stability [8,9]. Polycarbazole, polypyrrole, polytriphenylamine, polythiophene, polyaniline, poly(3,4-(2,2-diethylpropylenedioxy)thiophene) (PProDOT-Et_2_), and poly(3,4-ethylenedioxythiophene) are extensively used as conjugated polymers in electrochromic devices [10,11,12]. Among the various conjugated polymers, PProDOT-Et_2_ has shown great properties as a cathodically coloring material in electrochromic devices [13], and polycarbazoles were reported as anodically coloring materials in electrochromic devices [14] due to their high thermal stability, good optical properties, and interesting electroactive properties. Moreover, polycarbazoles can be easily functionalized at their *N*-, (2,7-), or (3,6-) positions [14,15,16,17,18]. Accordingly, thiophene- and carbazole-based conjugated polymers are employed to efficiently fabricate electroactive polymeric films for potential applications in electronic and optoelectronic devices. Moreover, ionic liquids (ILs) are salts which are liquid at a temperature of less than 100 °C, and are composed of organic cations and inorganic (or organic) anions. ILs show wide electrochemical potential windows, low or negligible volatility, high conductivity, and high thermal stability [19,20,21]. These properties make IL-based electrolytes ideal candidates for use as non-volatile electrolytes in ECDs.

In the present work, three polycarbazole derivatives (poly(3,6-di(2-thienyl)carbazole) (PDTC), poly(2,7-bis(carbazol-9-yl)-9,9-spirobifluorene) (PS2CBP), and poly(3,6-bis(*N*-carbazole)-*N*-ethylcarbazole) (PCEC)) were polymerized electrochemically, and their spectroelectrochemical and electrochromic properties were investigated. The incorporation of two thiophene units in the PDTC backbone results in a red shift of the absorption maxima and narrows the electrochemical band gap [22]. The incorporation of a spirobifluorene core in the PS2CBP backbone can provide specific properties, such as an elevated glass transition temperature and a stable decomposition temperature [23], and the spectroelectrochemical characterization of spirobifluorene core-based polycarbazole derivatives have not been reported so far. The ethylcarbazole core in the PCEC backbone is more electron donating than that of the spirobifluorene core in the PS2CBP backbone [24]. Furthermore, electrochromic devices were constructed using PDTC, PS2CBP, and PCEC films as anodic electrodes, poly(3,3-diethyl-3,4-dihydro-2H-thieno[3,4–b][1,4]dioxepine) (PProDOT-Et_2_) as cathodic electrodes, and an ionic liquid-based electrolyte as an electrochromic electrolyte. The electrochromic behaviors of PDTC film, PS2CBP film, PCEC film, a PDTC/PProDOT-Et_2_ ECD, a PS2CBP/PProDOT-Et_2_ ECD, and a PCEC/PProDOT-Et_2_ ECD were investigated systematically using spectroelectrochemistry, electrochromic switching, coloration efficiency, and colorimetry. The ECDs were also investigated using open circuit memory and long-term redox stability.

## 2. Materials and Methods

### 2.1. Materials and Electrochemical Synthesis

**Figure 1:**
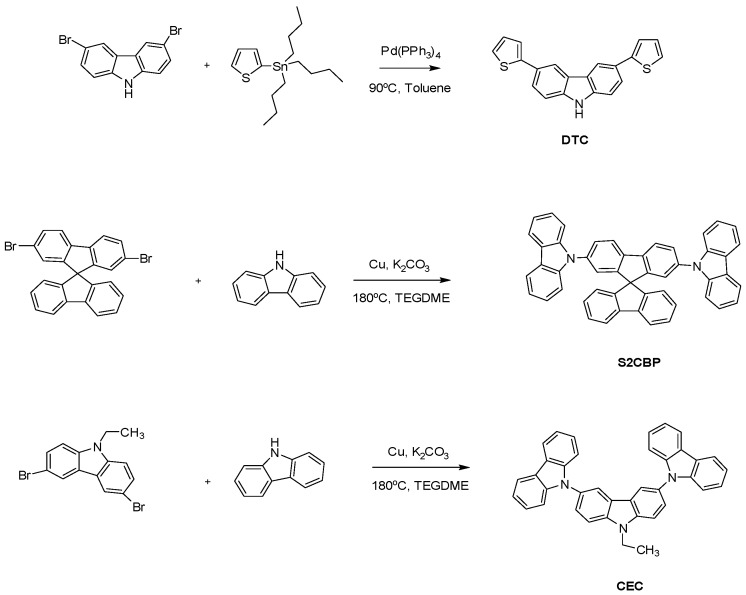
The synthetic routes of the three carbazole derivatives.

The reagents and compounds used in this study were purchased from Acros (Morris Plains, NJ, USA), TCI (Tokyo, Japan), and Aldrich (St. Louis, MO, USA), and used as received. The 3,3-diethyl-3,4-dihydro-2H-thieno [3,4–b][1,4]dioxepine (ProDOT-Et_2_) was synthesized according to previously published procedures [25]. The 1-ethyl-3-propylimidazolium bromide ([EPI^+^][Br^−^]) and 1-ethyl-3-propylimidazolium bis(trifluoromethanesulfonyl)imide ([EPI^+^][TFSI^−^]) were also synthesized based on previous procedures [26,27]. The synthetic routes of DTC, S2CBP, and CEC are shown in Figure 1. The PDTC, PS2CBP, and PCEC films were either prepared potentiostatically at 0.9 V, 1.2 V, and 1.1 V, respectively, on Indium Tin Oxide (ITO) glass electrodes with a charge density of 20 mC cm^−2^, or prepared potentiodynamically at a scan rate of 100 mV s^−1^ for 20 cycles. The onset potential of oxidation was determined from the first cyclic voltammogram (CV) cycle. The electrochromic behaviors were captured using a standard illuminant D65 light source.

#### 2.1.1. Synthesis of 3,6-Di(2-thienyl)carbazole (DTC)

A mixture of 2-(tributylstannyl)thiophene (2.50 g, 6.7 mmol), 3,6-dibromocarbazole (0.98 g, 3 mmol), 20 mL toluene, and 75 mg tetrakis(triphenylphosphine)palladium(0) was stirred at 90 °C under Argon for 48 h. Afterward, the mixture was filtered and the toluene was evaporated. The remaining crude product was purified using column chromatography (silica gel, eluent: dichloromethane/hexane mixture) to give the desired DTC. Yield: 61%. ^1^H-NMR (400 MHz, DMSO-*d*_6_): *δ* 11.46 (s, 1H, NH), 8.55, (s, 2H, carbazole-H), 7.72–7.70 (m, 2H, carbazole-H), 7.53–7.47 (m, 6H, carbazole-H and Th-H), 7.16–7.13 (m, 2H, Th-H). The ^1^H-NMR spectrum of DTC is displayed in Figure S1 (in supplementary information). Elemental analysis: Calculated (Elem. Anal. Calcd.) for C_20_H_13_NS_2_: C, 72.47%; H, 3.95%; N, 4.23%. Found: C, 72.35%; H, 3.88%; N, 4.16%.

#### 2.1.2. Synthesis of 2,7-Bis(carbazol-9-yl)-9,9-spirobifluorene (S2CBP)

In a round bottom flask were added 2,7-dibromo-9,9′-spirobifluorene (4.74 g, 10 mmol), carbazole (6.68 g, 40 mmol), potassium carbonate (5.53 g, 40 mmol), Cu powder (1.60 g, 25 mmol), and 20 mL triethylene glycol dimethyl ether (TEGDME), and the mixture was stirred at 180 °C under an Argon atmosphere for 36 h. TEGDME was evaporated using a rotary evaporator under an oil bath, and the reaction mixture was purified using column chromatography (silica gel, eluent: dichloromethane/hexane mixture). The recrystallization of the crude product from dimethylformamide gives S2CBP. Yield: 65%. ^1^H-NMR (400 MHz, DMSO-*d*_6_): *δ* 8.48 (d, 2H, carbazole-H), 8.19 (d, 4H, carbazole-H), 7.97 (d, 2H, fluorene-H), 7.78 (d, 2H, fluorene-H), 7.43 (d, 1H, carbazole-H), 7.42 (d, 1H, carbazole-H), 7.33 (dd, 4H, carbazole-H and fluorene-H), 7.28 (dd, 2H, fluorene-H), 7.23 (dd, 4H, carbazole-H and fluorene-H), 7.16 (d, 4H, carbazole-H), 6.98 (d, 2H, fluorene-H), 6.79 (s, 2H, fluorene-H). The ^1^H-NMR spectrum of S2CBP is displayed in Figure S2 (in supplementary information). Elem. Anal. Calcd. for C_49_H_30_N_2_: C, 90.99%; H, 4.68%; N, 4.33%. Found: C, 90.85%; H, 4.68%; N, 4.26%.

#### 2.1.3. Synthesis of 3,6-Bis(*N*-carbazolyl)-*N*-ethylcarbazole (CEC)

In a round bottom flask were added 3,6-dibromo-9-ethylcarbazole (4.94 g, 14 mmol), carbazole (9.36 g, 56 mmol), potassium carbonate (7.74 g, 56 mmol), Cu powder (2.30 g, 36 mmol), and 20 mL triethylene glycol dimethyl ether, and the mixture was stirred at 180 °C under an Argon atmosphere for 36 h. TEGDME was evaporated using a rotary evaporator under an oil bath, and the reaction mixture was purified using column chromatography (silica gel, eluent: dichloromethane/hexane mixture). The recrystallization of the crude product using dimethylformamide gives CEC. Yield: 58%. ^1^H-NMR (400 MHz, DMSO-*d*_6_): *δ* 8.56 (s, 2H, H_i_), 8.26 (d, 4H, H_h_), 7.99 (d, 2H, H_g_), 7.70 (d, 2H, H_f_), 7.42–7.40 (m, 4H, H_e_), 7.35–7.34 (m, 4H, H_d_), 7.26–7.28 (m, 4H, H_c_), 4.69 (q, 2H, H_b_), 1.51 (t, 3H, H_a_). The ^1^H-NMR spectrum of CEC is displayed in Figure S3 (in supplementary information). Elem. Anal. Calcd. for C_38_H_27_N_3_: C, 86.83%; H, 5.18%; N, 7.99%. Found: C, 86.75%; H, 5.10%; N, 7.96%.

### 2.2. Instrumentation and Measurements

The electrochemical behaviors of the PDTC, PS2CBP, and PCEC films coated on the ITO electrodes were characterized using a CHI6081E electrochemical analyzer (CH Instruments, Austin, TX, USA). The sheet resistance of ITO glass (AimCore Technology Co., Ltd., Hsinchu, Taiwan) is below 15 Ω/sq. The spectroelectrochemical properties of the PDTC film, the PS2CBP film, the PCEC film, a PDTC/PProDOT-Et_2_ ECD, a PS2CBP/PProDOT-Et_2_ ECD, and a PCEC/PProDOT-Et_2_ ECD were characterized using a V-670 JASCO UV-Visible spectrophotometer set to record in situ UV-Visible spectra photometer (JASCO International Co., Ltd., Tokyo, Japan). The chromaticity values of the polymer films and ECDs were calculated according to previous procedures [28].

### 2.3. Construction of ECDs

An electrochromic electrolyte was prepared according to the method described in our previous work [29]. The electrochromic electrolyte is an ionic liquid/polymer composite electrolyte. The ionic liquid and polymer are [EPI^+^][TFSI^−^] and poly(vinylidene fluoride-*co*-hexafluoropropylene) (PVdF-HFP), respectively. The anodic coloring PDTC, PS2CBP, and PCEC films were electrodeposited onto ITO glasses potentiostatically at 0.9 V, 1.2 V, and 1.1 V, respectively, whereas cathodic coloring PProDOT-Et_2_ film was electrodeposited onto ITO-coated glasses potentiostatically at 1.4 V. The anodic polymer film and PProDOT-Et_2_ film were separated by an ionic liquid/polymer composite electrolyte.

## 3. Results and Discussion

### 3.1. Electrochemical Polymerization of Polymer Films

**Figure 2:**
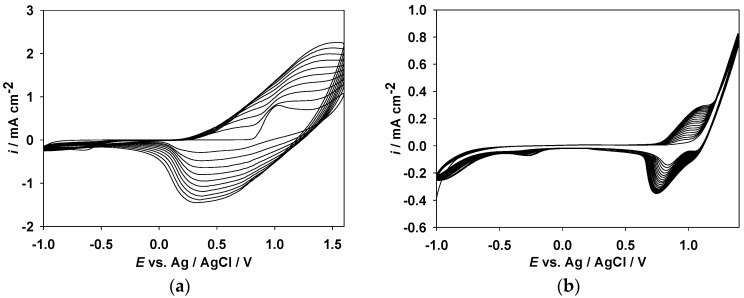

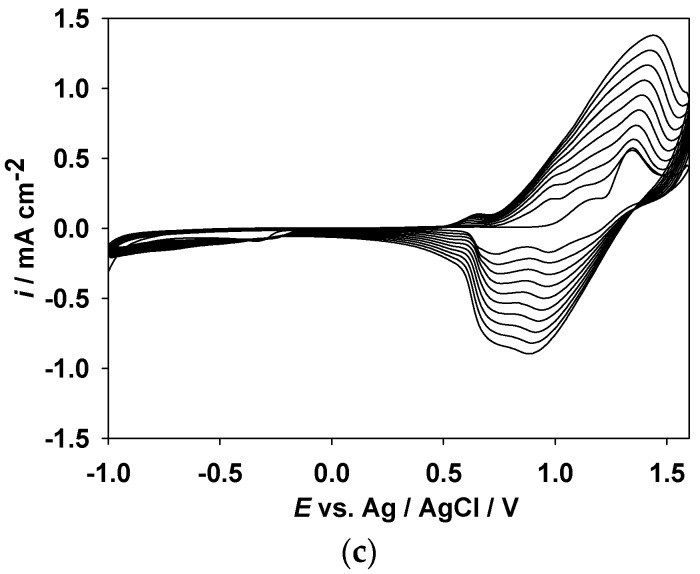
Cyclic voltammograms (CVs) of 2 mM (**a**) DTC, (**b**) S2CBP, and (**c**) CEC in ACN/DCM solution (1:1, *v*/*v*) containing 0.1 M LiClO_4_ at a scan rate of 100 mV s^−1^ on an ITO working electrode.

The polymer films can be prepared by a potentiodynamic method. The cyclic voltammograms (CVs) of neat DTC, S2CBP, and CEC monomers in an acetonitrile/dichloromethane (ACN/DCM) solution (1:1, *v*/*v*) containing 0.1 M LiClO_4_ are shown in Figure 2. As the cyclic voltammetric scan continued, the peak current intensity of Figure 2a–c increased gradually, demonstrating that the PDTC, PS2CBP, and PCEC films were electropolymerized on the surface of the ITO working electrode. The schemes for the electrochemical polymerization of PDTC, PS2CBP, and PCEC are shown in Figure S4 (in supplementary information).

The onset potentials of the PDTC, PS2CBP, and PCEC films are 0.82, 0.99, and 0.94 V, respectively. The PDTC film shows a lower onset potential than those of the PS2CBP and PCEC films, which can be attributed to the incorporation of two thiophene units at the 3,6-positions of the carbazole unit that gives rise to an obvious aromatic conjugation after electrochemical polymerization. Consequently, the onset potential of the PDTC film slightly shifts to low potential [30]. Moreover, the PCEC film shows a lower onset potential than that of the PS2CBP film, which can be ascribed to the fact that CEC contains one additional carbazole unit compared to the S2CBP unit [24]. The oxidation peaks of PDTC, PS2CBP, and PCEC as displayed in Figure 2 appear at 1.02, 1.21, and 1.32 V, respectively, whereas the reduction peaks of PDTC, PS2CBP, and PCEC are located at 0.31, 0.73, and 0.87 V, respectively.

**Figure 3:**
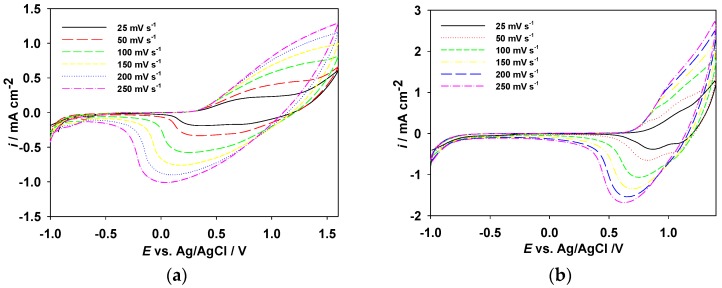

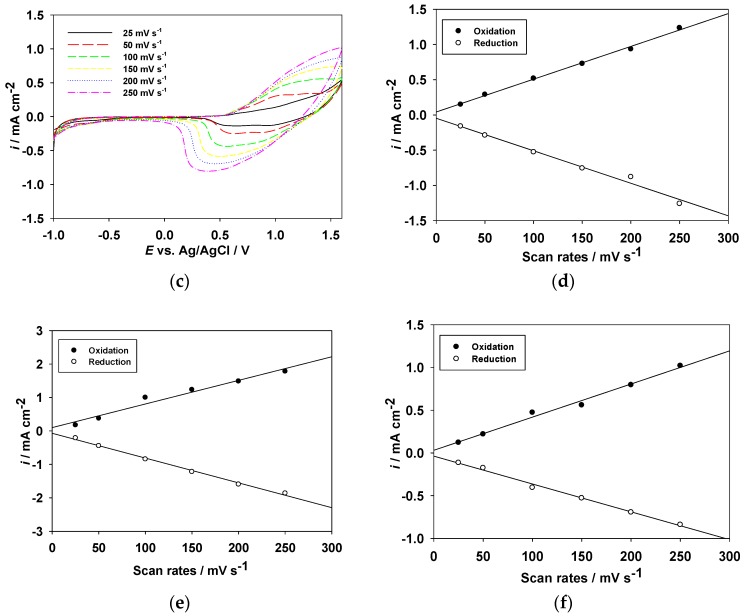
CV curves of (**a**) PDTC, (**b**) PS2CBP, and (**c**) PCEC films at various scan rates between 25 and 250 mV s^−1^ in [EPI^+^][TFSI^−^] solution, and the relationship between peak current density vs. scan rate of (**d**) PDTC, (**e**) PS2CBP, and (**f**) PCEC films in [EPI^+^][TFSI^−^] solution.

Figure 3a–c shows the CV plots of the PDTC, PS2CBP, and PCEC films, respectively, at different scan rates in [EPI^+^][TFSI^−^] solution, and the relationships between peak current density and the scan rate of the PDTC, PS2CBP, and PCEC films are shown in Figure 3d–f, respectively. The anodic and cathodic peak current density values increase linearly with increasing scan rate as displayed in Figure 3d–f, indicating that the oxidation and reduction processes are non-diffusion limited [31].

### 3.2. Electrochromic Properties of the PDTC, PS2CBP, and PCEC Films

**Figure 4:**
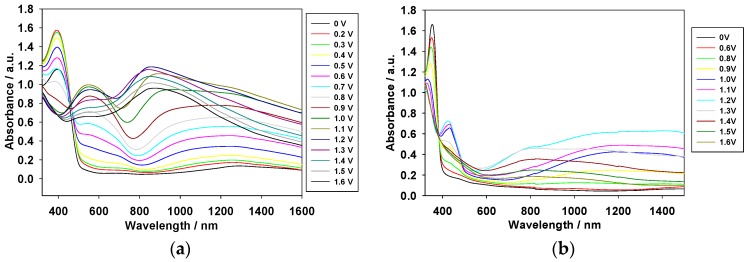

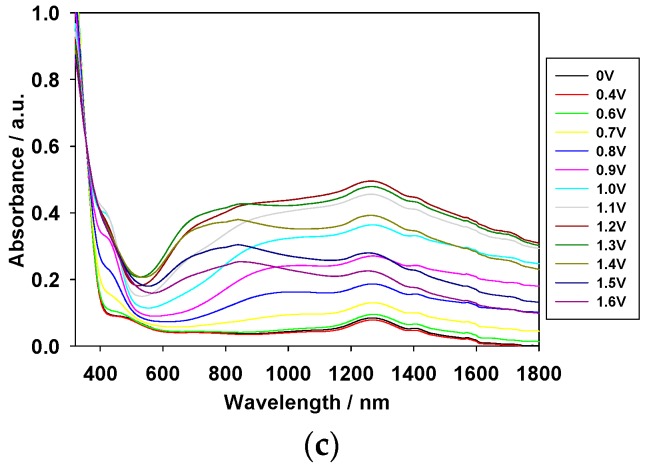
Spectroelectrochemical spectra of (**a**) PDTC; (**b**) PS2CBP; and (**c**) PCEC films on ITO electrodes at different applied potentials in [EPI^+^][TFSI^−^] solution.

The absorption spectra of the PDTC, PS2CBP, and PCEC films coated on ITO glass electrodes were investigated in [EPI^+^][TFSI^−^] solution at various potentials. As shown in Figure 4, the peaks of the PDTC and PS2CBP films in the neutral state were located at 406 nm and 354 nm, respectively, and the shoulder of the PCEC film in the neutral state was located at 415 nm. These peaks could be assigned to the π–π* transition of the PDTC, PS2CBP, and PCEC films in [EPI^+^][TFSI^−^] solution. In the PDTC film, the oxidation begins at about 0.5 V, the peak at around 406 nm decreases gradually, and the charge carrier bands appear at around 600 nm and 900 nm. The formation of the charge carrier bands can be ascribed to the evolution of polaron and bipolaron bands [32].

Under similar conditions, the oxidation of the PS2CBP film occurs at about 0.6 V, the peaks decrease gradually at around 350 nm, and increase gradually at around 430 nm and 1200 nm. Moreover, the oxidation of the PCEC film takes place at around 0.7 V, and the charge carrier bands at around 800 and 1300 nm increase gradually from 0.7 to 1.3 V. Table 1 shows the photos of the PDTC, PS2CBP, and PCEC films in [EPI^+^][TFSI^−^] solution at various potentials. The PDTC, PS2CBP, and PCEC films show reversible electrochromic behaviors in their redox states. The PDTC film was yellowish-green in the neutral state (0 V), green in the intermediate state (1.0 V), iron gray in the oxidized state (1.2 V), and bluish-purple in highly oxidized states (1.4 V). Similarly, the PS2CBP film shows a distinct color transition with four various colors (gray at 0 V, grayish-green at 1.0 V, moss green at 1.2 V, and foliage green at 1.4 V), and the PCEC film displays four various colors from neutral to highly oxidized states (silver gray at 0 V, cobalt green at 1.0 V, celadon green at 1.2 V, and peacock green at 1.4 V). The colorimetric values (L*, a*, b*) of the PDTC, PS2CBP, and PCEC films measured at various potentials in [EPI^+^][TFSI^−^] solution are listed in Table 2, and the CIE (Commission Internationale de I’Eclairage) chromaticity diagrams of PDTC, PS2CBP, and PCEC films in neutral and oxidation state are displayed in Figure S5 (in supplementary information). The L*, a*, b* of the PS2CBP film at 0 V was 89.71, 0.30, and 7.51, respectively. The a* values of the PS2CBP film convert from positive to negative at 0.8–1.4 V, indicating that the color of the PS2CBP film turns from light gray to green in the oxidation state.

The optical band gap (*E*_g_) of the PDTC, PS2CBP, and PCEC films can be calculated according to the Planck equation (*E*_g_ = 1241 (eV·nm)/*λ*_onset_ (nm)) [33,34,35], and they are 2.45, 3.06, and 3.00 eV, respectively. The *E*_g_ of the PS2CBP film is comparable to that of the PCEC film, implying that the incorporation of a spirofluorene (or carbazole) unit between two carbazole groups does not influence *E*_g_ significantly. However, the PDTC film shows a lower *E*_g_ value than those of the PS2CBP and PCEC films, which can be ascribed to the fact that the incorporation of two thiophene units at the 3,6-positions of the carbazole group diminishes the *E*_g_ value significantly.

The highest occupied molecular orbital energy levels (*E*_HOMO_) of the PDTC, PS2CBP, and PCEC films were calculated using the formula [36]:

*E*_HOMO_ = −e(*E*_onset_ + 4.8) (vs. vacuum) (1)

where *E*_onset_ is the onset oxidation potentials corrected using an internal standard redox ferrocene/ferrocinium couple. The lowest unoccupied molecular orbital energy levels (*E*_LUMO_) of the PDTC, PS2CBP, and PCEC films were calculated by subtracting the optical band gap from *E*_HOMO_ [37]. The *E*_HOMO_ of PDTC, PS2CBP, and PCEC are −5.14, −5.31, and −5.26 eV, respectively, and the *E*_LUMO_ of PDTC, PS2CBP, and PCEC are −2.69, −2.25, and −2.26 eV, respectively.

**Figure 5:**
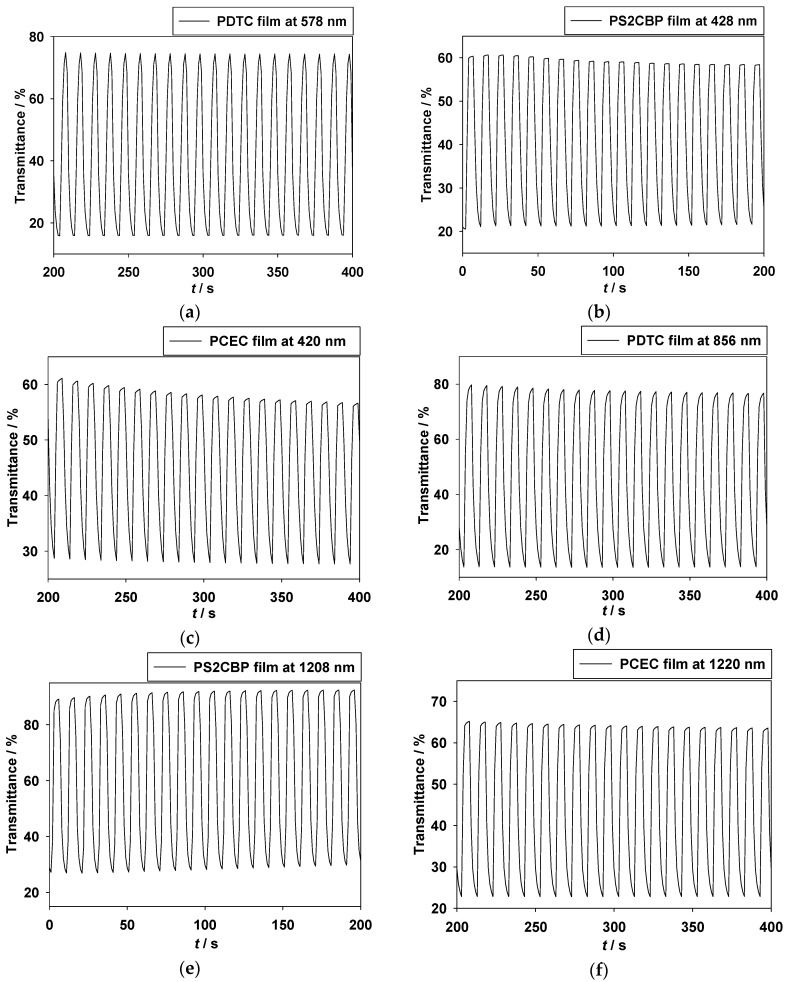
In situ transmittance of (**a**) the PDTC film at 578 nm under a voltage interval between 0 V and +1.0 V; (**b**) PS2CBP film at 428 nm under a voltage interval between 0 V and +1.2 V; (**c**) the PCEC film at 420 nm under a voltage interval between 0 V and +0.9 V; (**d**) the PDTC film at 856 nm under a voltage interval between 0 V and +1.0 V; (**e**) the PS2CBP film at 1208 nm under a voltage interval between 0 V and +1.2 V; (**f**) the PCEC film at 1220 nm under a voltage interval between 0 V and +1.2 V as a function of time in [EPI^+^][TFSI^−^] solution, the time interval is 5 s.

A cyclic potential-step method was used to determine the electrochromic switching of conducting polymer films [38]. The electrochromic switching of PDTC, PS2CBP, and PCEC films in [EPI^+^][TFSI^−^] solution was stepped by repeated potential between reduction and oxidation states with a time interval of 5 s. Figure 5 exhibits the transmittance–time profiles of the PDTC film at 578 and 856 nm, the PS2CBP film at 428 and 1208 nm, and the PCEC film at 420 and 1220 nm.

The coloration switching time (*τ*_c_) and bleaching switching time (*τ*_b_) of the PDTC, PS2CBP, and PCEC films in [EPI^+^][TFSI^−^] solution are listed in Table 3; the *τ*_c_ and *τ*_b_ are determined at 90% of full-transmittance change (*T*_90%_). The optical switching time of the PDTC film in [EPI^+^][TFSI^−^] solution was found to be 2.04 and 1.64 s at 578 and 856 nm, respectively, from the bleaching to coloring state at the 50th cycle, and 2.01 and 1.69 s at 578 and 856 nm, respectively, from the coloring to bleaching state at the 50th cycle. The *τ*_c_ and *τ*_b_ of the PS2CBP film in [EPI^+^][TFSI^−^] solution at 1208 nm were found to be 2.16 and 1.83 s, respectively, at the 100th cycle, and the *τ*_c_ and *τ*_b_ of the PCEC film in [EPI^+^][TFSI^−^] solution at 1220 nm were found to be 1.90 and 1.63 s, respectively, at the 100th cycle.

For the switching times of the PS2CBP and PCEC films at different cycles, the *τ*_c_ and *τ*_b_ of the PS2CBP film in [EPI^+^][TFSI^−^] solution at 1208 nm were 2.24 and 1.98 s, respectively, at the first cycle, and 2.16 and 1.83 s, respectively, at the 100th cycles, and the *τ*_c_ and *τ*_b_ of the PCEC film in [EPI^+^][TFSI^−^] solution at 1220 nm were 1.99 and 1.72 s, respectively, at the first cycle, and 1.90 and 1.63 s, respectively, at the 100th cycles, indicating that the *τ*_c_ and *τ*_b_ of PS2CBP and PCEC films do not show significant change at high switching cycles when we employ [EPI^+^][TFSI^−^] as a supporting electrolyte.

The optical contrast (Δ*T*%) is the most important parameter for electrochromic applications [39]. The Δ*T*_max_ of the PDTC film at 578 and 856 nm is 58.79% and 66.04%, respectively, in [EPI^+^][TFSI^−^] solution, and the Δ*T*_max_ of the PS2CBP film at 428 nm and 1208 nm is 39.83% and 63.56%, respectively, in [EPI^+^][TFSI^−^] solution, implying that the incorporation of the DTC unit gives rise to a higher Δ*T*_max_ than that of the S2CBP unit. The Δ*T*_max_ of the PCEC film at 420 nm and 1220 nm is 32.41% and 42.36%, respectively, in [EPI^+^][TFSI^−^] solution. The Δ*T*_max_ of the PCEC film is lower than that of the PS2CBP film, indicating that two carbazole units linked by a spirobifluorene group leads to a higher Δ*T*_max_ than that of two carbazole units linked by an *N*-ethylcarbazole group. Among these polymer films, the PDTC film shows the highest Δ*T*_max_ (66.04%), at 856 nm in [EPI^+^][TFSI^−^] solution. The Δ*T*_max_ of the PS2CBP film is higher than that reported for poly(4-(3,6-di(thiophen-2-yl)-9H-carbazol-9-yl)-phenyl-methanone) (PTCPM) (Δ*T*_max_ = 41% at 1100 nm) [29], and is comparable to that reported for poly(2,8-di(carbazol-9-yl)dibenzothiophene)(PSCZ) (Δ*T*_max_ = 61% at 762 nm) [40]. The Δ*T*_max_ of the PCEC film is higher than those reported for PTCPM (Δ*T*_max_ = 41% at 1100 nm) and poly(3,6-di(carbazol-9-yl)-*N*-(4-methoxyphenyl) carbazole)(PPhCz-2Cz) (Δ*T*_max_ = 37% at 741 nm) [29,41], whereas the PCEC film shows a lower Δ*T*_max_ than that reported for PSCZ (Δ*T*_max_ = 61% at 762 nm) [40].

The coloration efficiency (CE, *η*) is defined as the change in the optical absorbance per unit of inserted charge (*Q*_d_) in electrochromic materials and ECDs. CE can be calculated using the following equation at a given wavelength [42]:

ΔOD = log(*T*_b_/T_c_) (2)

CE = ΔOD/*Q*_d_ (3)

where ΔOD indicates the change of the optical density at a specific wavelength. *T*_b_ and *T*_c_ are defined as the transmittance of the bleaching state and coloring state, respectively. The calculated *η*_max_ of the PDTC film is 201.61 cm^2^ C^−^^1^ at 578 nm and 167.83 cm^2^ C^−^^1^ at 856 nm; the *η*_max_ of the PS2CBP film is 138.09 cm^2^ C^−^^1^ at 428 nm and 151.70 cm^2^ C^−^^1^ at 1208 nm; and the *η*_max_ of the PCEC film is 293.91 cm^2^ C^−^^1^ at 420 nm and 214.07 cm^2^ C^−^^1^ at 1220 nm. The PDTC, PS2CBP, and PCEC films in [EPI^+^][TFSI^−^] solution show higher *η* than those reported for PTCPM (*η* = 110.48 cm^2^ C^−1^ at 1100 nm) [29], PSCZ (*η* = 45 cm^2^ C^−1^ at 762 nm) [40], and PPhCz-2Cz (*η* = 56 cm^2^ C^−1^ at 741 nm) [41]. This may be attributed to that fact that an ionic liquid solution is employed as an electrochromic electrolyte in this study.

### 3.3. Spectroelectrochemistry of Electrochromic Devices

**Figure 6:**
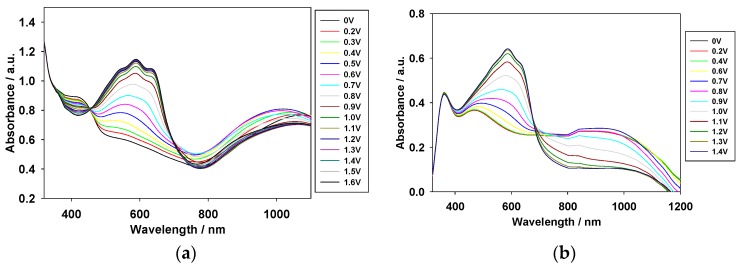

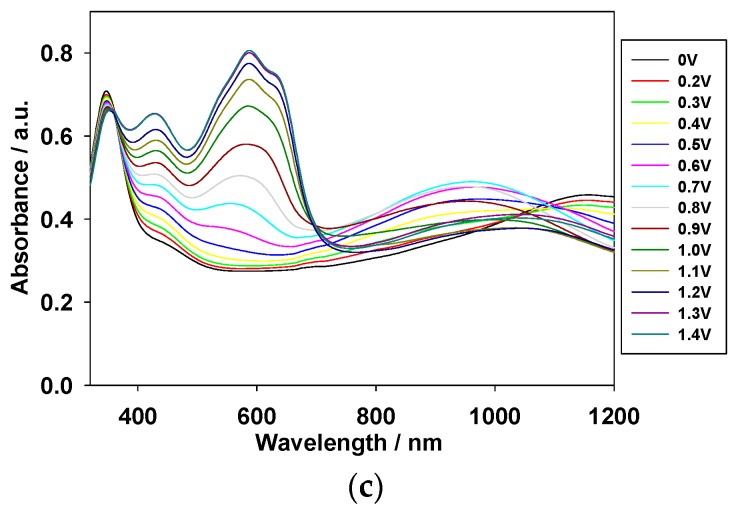
Spectroelectrochemical spectra of (**a**) PDTC/PProDOT-Et_2_; (**b**) PS2CBP/PProDOT-Et_2_; and (**c**) PCEC/PProDOT-Et_2_ ECDs at applied various potentials.

Dual-type ECDs were constructed using two complementary electrochromically active layers. Dual-type ECDs sometimes exhibit a higher electrochromic (EC) contrast in a wider visible range than those of single-type ECDs. Dual-type ECDs comprise anodically coloring material (PDTC, PS2CBP, or PCEC), cathodically coloring material (PProDOT-Et_2_), and ionic liquid-PVdF-HFP. Composite electrolytes were fabricated and their spectroelectrochemical properties were characterized. The spectroelectrochemical spectra of the PDTC/PProDOT-Et_2_, PS2CBP/PProDOT-Et_2_, and PCEC/PProDOT-Et_2_ ECDs are displayed in Figure 6a–c, respectively. As displayed in Figure 6a, the PDTC/PProDOT-Et_2_ ECD shows a shoulder at around 415 nm at 0 V, which can be attributed to the π–π* transition of the PDTC film in the reduction state. In this circumstance, the PProDOT-Et_2_ film was light blue in its oxidation state, and the PDTC/PProDOT-Et_2_ ECD was olive green at 0 V. However, the π–π* transition absorption of the PDTC film diminished and a new absorption band at 590 nm emerged gradually with increasing potential. The PDTC/PProDOT-Et_2_ ECD was dark gray at 1.0 V, prussian blue at 1.2 V, and midnight blue at 1.4 V. In a similar situation, the PS2CBP/PProDOT-Et_2_ ECD was silver gray at 0 V, cornflower blue at 1.0 V, and salvia blue at 1.2 V and 1.4 V, and the PCEC/PProDOT-Et_2_ ECD was gray at 0 V, dark mineral blue at 1.0 V, and slate blue at 1.2 V and 1.4 V. The colorimetric values and CIE chromaticity values of the PDTC/PProDOT-Et_2_, PS2CBP/PProDOT-Et_2_, and PCEC/PProDOT-Et_2_ ECDs are listed in Table 4. Moreover, the CIE chromaticity diagrams of the PDTC/PProDOT-Et_2_, PS2CBP/PProDOT-Et_2_, and PCEC/PProDOT-Et_2_ ECDs at bleaching and coloring states are shown in Figure S6 (in supplementary information).

**Figure 7:**
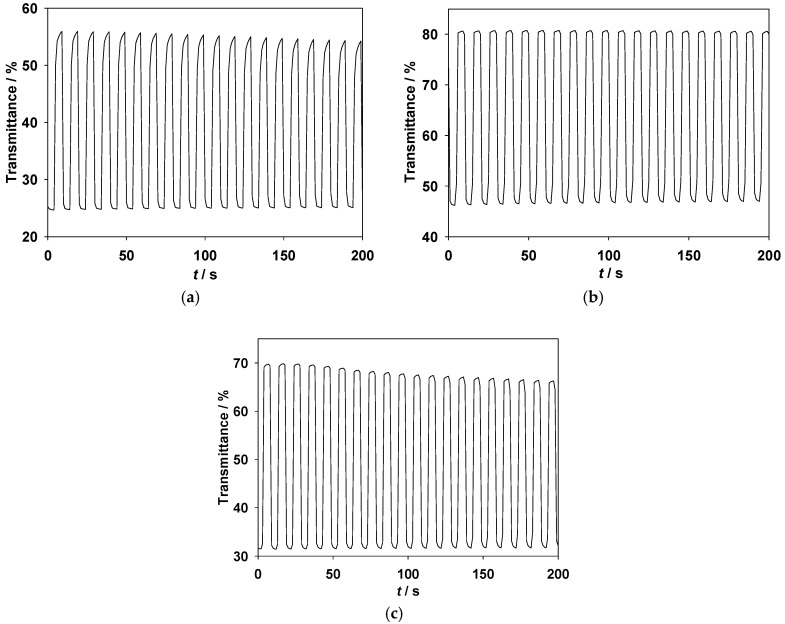
In situ transmittance of (**a**) the PDTC/PProDOT-Et_2_ ECD at 592 nm as a function of time under a voltage interval between −0.4 V and +1.0 V; (**b**) the PS2CBP/PProDOT-Et_2_ ECD at 590 nm under a voltage interval between −0.4 V and 1.4 V; (**c**) the PCEC/PProDOT-Et_2_ ECD at 586 nm under a voltage interval between 0 V and 1.4 V.

The transmittance–time profiles of the PDTC/PProDOT-Et_2_, PS2CBP/PProDOT-Et_2_, and PCEC/PProDOT-Et_2_ ECDs are shown in Figure 7a–c, respectively, and the Δ*T*_max_, ΔOD, and *η*_max_ of the PDTC/PProDOT-Et_2_, PS2CBP/PProDOT-Et_2_, and PCEC/PProDOT-Et_2_ ECDs are summarized in Table 5. The Δ*T*_max_ of the PDTC/PProDOT-Et_2_, PS2CBP/PProDOT-Et_2_, and PCEC/PProDOT-Et_2_ ECDs are 31.27% at 592 nm, 34.45% at 590 nm, and 38.25% at 586 nm, respectively. On the other hand, the *η*_max_ of the PDTC/PProDOT-Et_2_, PS2CBP/PProDOT-Et_2_, and PCEC/PProDOT-Et_2_ ECDs are estimated to be 345.19 cm^2^ C^−^^1^ at 592 nm, 256.12 cm^2^ C^−^^1^ at 590 nm, and 369.85 cm^2^ C^−^^1^ at 586 nm, respectively. The Δ*T*_max_ and *η* of the PCEC/PProDOT-Et_2_ ECD are larger than those of poly(4,4’-di(*N*-carbazoyl)biphenyl-*co*-2,2′-bithiophene)/PEDOT (Δ*T*_max_ = 28.6%, *η*_max_ = 234 cm^2^ C^−^^1^ at 700 nm) and poly(9H-carbazol-9-ylpyrene)/PEDOT (Δ*T*_max_ = 23%, *η*_max_ = 290 cm^2^ C^−^^1^ at 623 nm) [43,44]. However, the PCEC/PProDOT-Et_2_ ECD shows a lower Δ*T*_max_% and *η*_max_ than those reported for PCBTD/PEDOT (Δ*T*_max_ = 49.4% at 620 nm, *η*_max_ = 1728 cm^2^ C^−^^1^) and P(BCz1-*co*-Inc2)/PProDOT-Et_2_ ECDs (Δ*T*_max_ = 42%, *η*_max_ = 634 cm^2^ C^−^^1^ at 587 nm) [45,46].

The *τ*_c_ and *τ*_b_ of the ECDs estimated at different double-step potential cycles are summarized in Table 3. The *τ*_c_ of the PDTC/PProDOT-Et_2_, PS2CBP/PProDOT-Et_2_, and PCEC/PProDOT-Et_2_ ECDs are 0.99, 1.06, and 0.98 s at the 100th cycles, respectively, and the *τ*_b_ of the PDTC/PProDOT-Et_2_, PS2CBP/PProDOT-Et_2_, and PCEC/PProDOT-Et_2_ ECDs are 0.97, 1.00, and 0.90 s at the 100th cycles, respectively. The *τ*_c_ and *τ*_b_ of the PDTC/PProDOT-Et_2_, PS2CBP/PProDOT-Et_2_, and PCEC/PProDOT-Et_2_ ECDs are shorter than those of the PDTC, PS2CBP, and PCEC films in an ionic liquid solution, indicating that the ECDs changed color faster from a bleaching to a coloring state than those of the PDTC, PS2CBP, and PCEC films in an ionic liquid solution.

**Figure 8:**
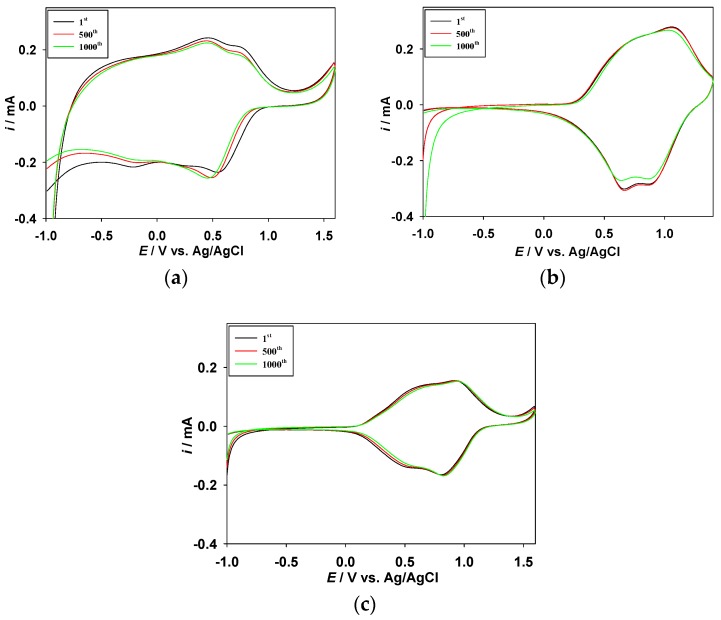
Cyclic voltammograms of (**a**) PDTC/PProDOT-Et_2_; (**b**) PS2CBP/PProDOT-Et_2_; and (**c**) PCEC/PProDOT-Et_2_ ECDs as a function of repeated scans at 100 mV s^−1^.

The long-term redox stability between the dedoped and doped states is an important parameter of ECDs [47]. The redox stability of the PDTC/PProDOT-Et_2_, PS2CBP/PProDOT-Et_2_, and PCEC/PProDOT-Et_2_ ECDs were measured by CV at potential range between −1.0 V and +1.6 V (or −1.0 V and +1.5 V) with a scan rate of 100 mV s^−1^. As shown in Figure 8, the PDTC/PProDOT-Et_2_, PS2CBP/PProDOT-Et_2_, and PCEC/PProDOT-Et_2_ ECDs exhibited 92%, 94%, and 95%, respectively, of electrochemical activity maintenance after the 500th cycle, and 89%, 92%, and 93%, respectively, of electrochemical activity maintenance after the 1000th cycle, implying that the PDTC/PProDOT-Et_2_, PS2CBP/PProDOT-Et_2_, and PCEC/PProDOT-Et_2_ ECDs have a reasonable environmental and redox stability.

**Figure 9:**
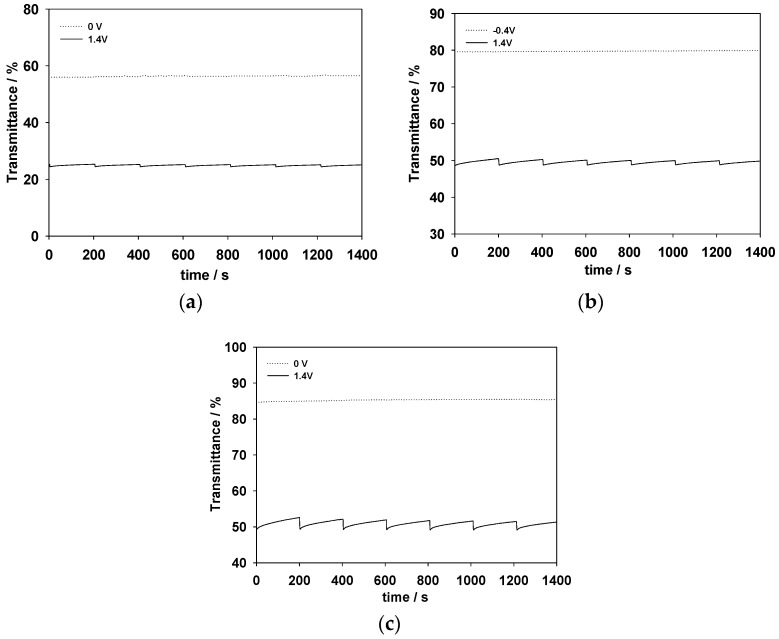
Open circuit stability of (**a**) PDTC/PProDOT-Et_2_ ECD monitored at 592 nm; (**b**) PS2CBP/PProDOT-Et_2_ ECD monitored at 590 nm; and (**c**) PCEC/PProDOT-Et_2_ ECD monitored at 586 nm.

Open circuit memory is another important effect for ECDs, as the effect denotes energy consumption during the operations of ECDs [48,49]. The open circuit memory for the PDTC/PProDOT-Et_2_, PS2CBP/PProDOT-Et_2_, and PCEC/PProDOT-Et_2_ ECDs were evaluated at ca. 590 nm with a function of time by applying potentials at a bleaching state (0 or −0.4 V) and at a coloring state (+1.4 V) for 1 s for each 200 s time interval. As shown in Figure 9, the PDTC/PProDOT-Et_2_, PS2CBP/PProDOT-Et_2_ and PCEC/PProDOT-Et_2_ ECDs exhibit a satisfactory optical memory effect at a coloring state (<5% transmittance change) and a bleaching state (transmittance variation is insignificant), demonstrating that the PDTC/PProDOT-Et_2_, PS2CBP/PProDOT-Et_2_ and PCEC/PProDOT-Et_2_ ECDs display good optical memory at both bleaching and coloring states.

## 4. Conclusions

DTC, S2CBP, and CEC were synthesized chemically and their corresponding homopolymers (PDTC, PS2CBP, and PCEC) were chemically synthesized using electrochemical polymerization. The PDTC, PS2CBP, and PCEC films show reversible electrochromic behaviors, and display a distinct color transition with four various colors in an ionic liquid solution. The PCEC film displays various colors from neutral to highly oxidized states (light gray at 0 V, cobalt green at 1.0 V, celadon green at 1.2 V, and peacock green at 1.4 V). Electrochromic switching investigations of the PDTC, PS2CBP, and PCEC films indicate that PS2CBP film has a high Δ*T*_max_ (Δ*T*_max_ = 63.56% at 1208 nm) and PCEC film has high coloration efficiency (*η*_max_ = 293.91 cm^2^ C^−^^1^ at 420 nm and 214.07 cm^2^ C^−^^1^ at 1220 nm) in an ionic liquid solution. ECDs were also fabricated by utilizing PDTC, PS2CBP, and PCEC as anodically coloring electrochromic materials and PProDOT-Et_2_ as a cathodically coloring electrochromic material. The PS2CBP/PProDOT-Et_2_ and PCEC/PProDOT-Et_2_ ECDs exhibit high Δ*T*_max_ (Δ*T*_max_ = 34.45% at 590 nm for PS2CBP/PProDOT-Et_2_ ECD and Δ*T*_max_ = 38.25% at 586 nm for PCEC/PProDOT-Et_2_ ECD) and high coloration efficiency (*η*_max_ = 256.12 cm^2^ C^−^^1^ at 590 nm for PS2CBP/PProDOT-Et_2_ ECD and *η*_max_ = 369.85 cm^2^ C^−^^1^ at 586 nm for PCEC/PProDOT-Et_2_ ECD). As a result, the PS2CBP/PProDOT-Et_2_ and PCEC/PProDOT-Et_2_ ECDs are promising candidates for electrochromic applications.

## Figures and Tables

**Table 1:** Electrochromic behaviors of the PDTC, PS2CBP, and PCEC films in [EPI^+^][TFSI^−^] solution and their corresponding electrochromic devices (ECDs) at a potential range from 0 V to +1.4 V.

Polymer Films and ECDs	0 V	1.0 V	1.2 V	1.4 V
PDTC film	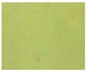	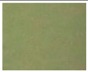	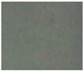	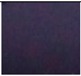
PS2CBP film	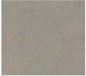	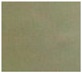	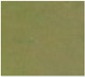	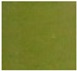
PCEC film	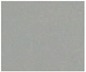	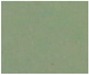	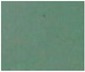	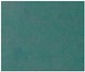
PDTC/PProDOT-Et_2_ ECD	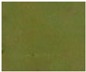	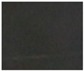	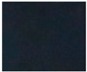	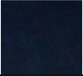
PS2CBP/PProDOT-Et_2_ ECD	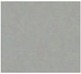	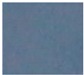	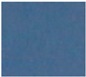	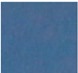
PCEC/PProDOT-Et_2_ ECD	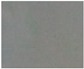	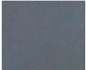	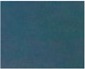	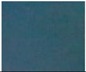

**Table 2:** The colorimetric values (L*, a*, b*) of the PDTC, PS2CBP, and PCEC films at different applied potentials in [EPI^+^][TFSI^−^] solution.

Polymer Films	*E*/V	L*	a*	b*
PDTC	0	87.23	−9.03	73.27
	0.8	51.93	−1.93	9.15
	1.0	40.99	8.39	−13.85
	1.2	41.62	6.2	−12.64
	1.4	45.89	2.8	−9.75
PS2CBP	0	89.71	0.3	7.51
	0.8	86.31	−0.97	15.64
	1.0	83.25	−6.74	36.93
	1.2	78.29	−11.76	32.53
	1.4	77.82	−1.2	14.99
	1.6	82.04	0.96	17.56
PCEC	0	94.61	1.18	4.37
	0.8	92.32	−3.15	13.78
	1.0	88.73	−8.42	21.07
	1.2	82.58	−11.22	10.04
	1.4	81.23	−8.44	8.55
	1.6	85.25	−4.96	13.79

**Table 3:** Color-bleach kinetics of the PDTC, PS2CBP, and PCEC films in [EPI^+^][TFSI^−^] solution and the ECDs.

Polymer Films and ECDs	*λ*_max_/nm	Cycle No.	Δ*T*/%	*τ*_c_/s	*τ*_b_/s
PDTC films in [EPI^+^][TFSI^−^]	578	1	58.79	1.91	1.93
		50	58.11	2.04	2.01
		100	58.02	2.07	2.04
	856	1	66.04	1.77	1.72
		50	61.77	1.64	1.69
		100	56.96	1.68	1.64
PS2CBP films in [EPI^+^][TFSI^−^]	428	1	39.83	1.85	1.80
		50	33.29	2.07	1.97
		100	27.95	2.08	1.95
	1208	1	63.56	2.24	1.98
		50	57.94	2.23	1.91
		100	50.64	2.16	1.83
PCEC films in [EPI^+^][TFSI^−^]	420	1	32.41	2.21	1.77
		50	28.27	2.24	1.74
		100	26.98	2.22	1.77
	1220	1	42.36	1.99	1.72
		50	40.15	1.96	1.66
		100	38.59	1.90	1.63
PDTC/PProDOT-Et_2_ ECD	592	1	31.27	0.96	0.99
		50	26.77	0.96	0.96
		100	24.85	0.99	0.97
PS2CBP/PProDOT-Et_2_ ECD	590	1	34.45	1.06	0.99
		50	32.67	1.04	0.99
		100	31.84	1.06	1.00
PCEC/PProDOT-Et_2_ ECD	586	1	38.25	1.01	0.96
		50	32.19	1.01	0.93
		100	29.90	0.98	0.90

**Table 4:** The colorimetric values (L*, a*, b*) of the PDTC/PProDOT-Et_2_, PS2CBP/PProDOT-Et_2_, and PCEC/PProDOT-Et_2_ ECDs at different applied potentials.

ECDs	*E*/V	L*	a*	b*
PDTC/PProDOT-Et_2_	0	47.59	0.46	12.09
	0.8	34.78	3.65	−7.51
	1.0	32.48	4.14	−11.21
	1.2	31.68	5.13	−12.89
	1.4	31.46	5.79	−13.69
PS2CBP/PProDOT-Et_2_	0	70.88	8.23	19.31
	0.8	60.51	0.89	0.57
	1.0	59.73	0.89	−1.1
	1.2	59.45	0.84	−2.04
	1.4	59.34	0.81	−2.84
PCEC/PProDOT−Et_2_	0	77.37	−1.62	6.39
	0.8	64.15	−0.22	0.56
	1.0	56.84	−2.66	−6.22
	1.2	52.94	−3.92	−8.46
	1.4	51.66	−4.82	−7.92

**Table 5:** Comparisons of the Δ*T*_max_ and *η*_max_ for various polymer films and ECDs.

Polymer Films and ECDs	*λ*/nm	*E*_g_/eV	Δ*T*_max_/%	ΔOD_max_/%	*η*_max_/cm^2^ C^−1^
PDTC	856	2.45	66.04	76.46	167.83
PS2CBP	1208	3.06	63.56	51.41	151.70
PCEC	1220	3.00	42.36	45.55	214.07
PTCPM [29]	1100	-	41	-	110.48
PSCz [40]	762	3.26	61	45.90	45
PPhCz-2Cz [41]	741	2.76	37	-	56
PDTC/PProDOT-Et_2_ ECD	592	-	31.27	35.55	345.19
PS2CBP/PProDOT-Et_2_ ECD	590	-	34.45	24.19	256.12
PCEC/PProDOT-Et_2_ ECD	586	-	38.25	34.52	369.85
P(CBP-*co*-BT)/PEDOT ECD [43]	700	-	28.6	-	234
PMCzP/PEDOT ECD [44]	623	-	23	-	290
PCBTD/PEDOT ECD [45]	620	-	49.4	-	1728
P(BCz1-*co*-Inc2)/PProDOT-Et_2_ ECD [46]	587	-	42	-	634

## Supplementary Materials

The following are available online at http://www.mdpi.com/2073-4360/9/7/284/s1, Figure S1: ^1^H NMR spectrum of DTC in DMSO-*d*_6_, Figure S2: ^1^H NMR spectrum of S2CBP in DMSO-*d*_6_, Figure S3: ^1^H NMR spectrum of CEC in DMSO-*d*_6_, Figure S4: The schemes for electrochemical polymerization of PDTC, PS2CBP, and PCEC, Figure S5: CIE chromaticity diagrams of PDTC, PS2CBP, and PCEC film in [EPI^+^][TFSI^–^] solution, Figure S6: CIE chromaticity diagrams of PDTC/PProDOT-Et_2_, PS2CBP/PProDOT-Et_2_, and PCEC/PProDOT-Et_2_ ECDs, Figure S7: Spectroelectrochemical spectra of PProDOT-Et_2_ film in [EPI^+^][TFSI^–^] solution, Table S1: The CIE chromaticity values of PDTC, PS2CBP, and PCEC films at different applied potentials in [EPI^+^][TFSI^–^], Table S2: The CIE chromaticity values of PDTC/PProDOT-Et_2_, S2CBP/PProDOT-Et_2_, and PCEC/PProDOT-Et_2_ ECDs at different applied potentials.
