# Supplementary material for: Three Carbazole-Based Polymers as Potential Anodically Coloring Materials for High-Contrast Electrochromic Devices
Source: Polymers (Basel). 2017 Jul 18;9(7):284. doi: 10.3390/polym9070284 (PMC6431872; doi:10.3390/polym9070284)
Supplement: Supplementary file 1 [file polymers-09-00284-s001.pdf]

polymers

MDPI

Supplementary Information

Three carbazole-based polymers as potential anodically coloring materials for high-contrast electrochromic devices

Yuh-Shan Su, Tzi-Yi Wu*

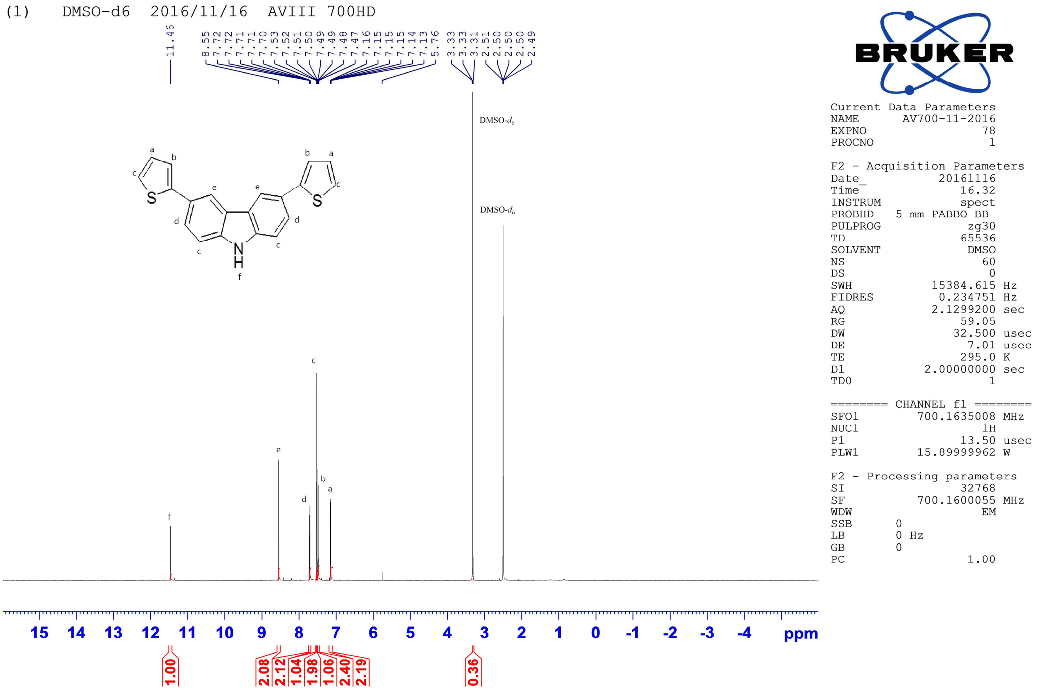

Fig. S1. ^{1}H NMR spectrum of DTC in DMSO-d_6.

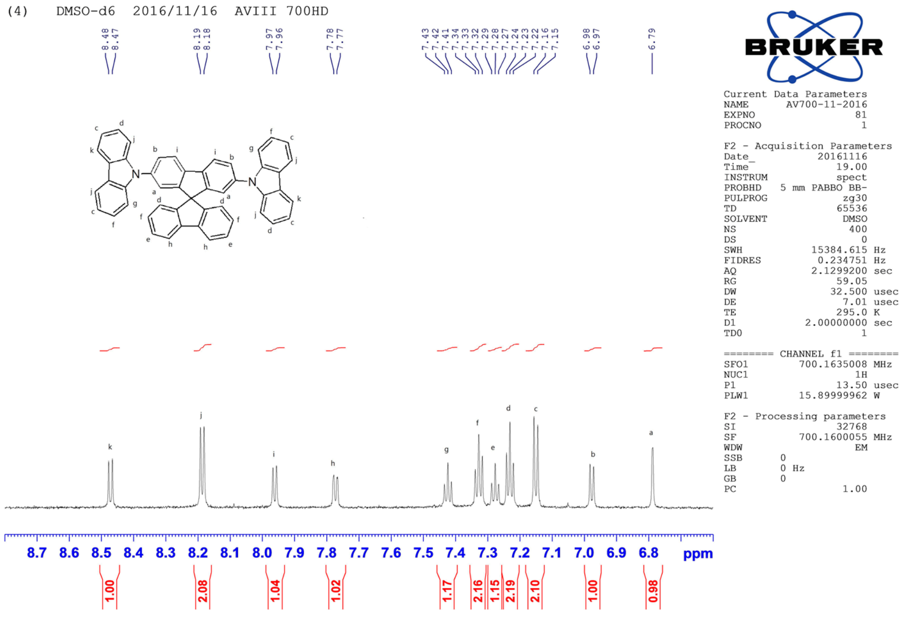

Fig. S2. ^{1}H NMR spectrum of S2CBP in DMSO-d_6.

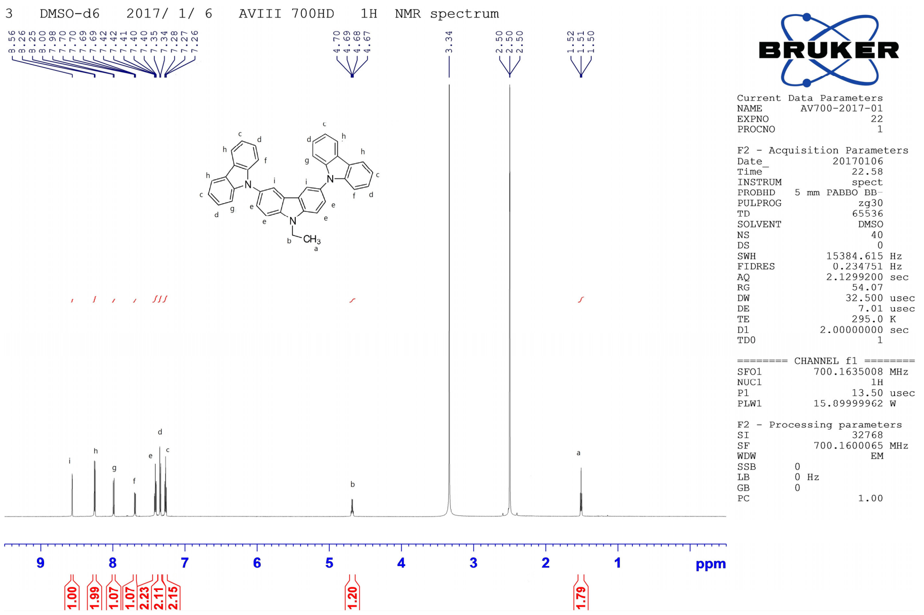

Fig. S3. ^{1}H NMR spectrum of CEC in DMSO-*d*6.

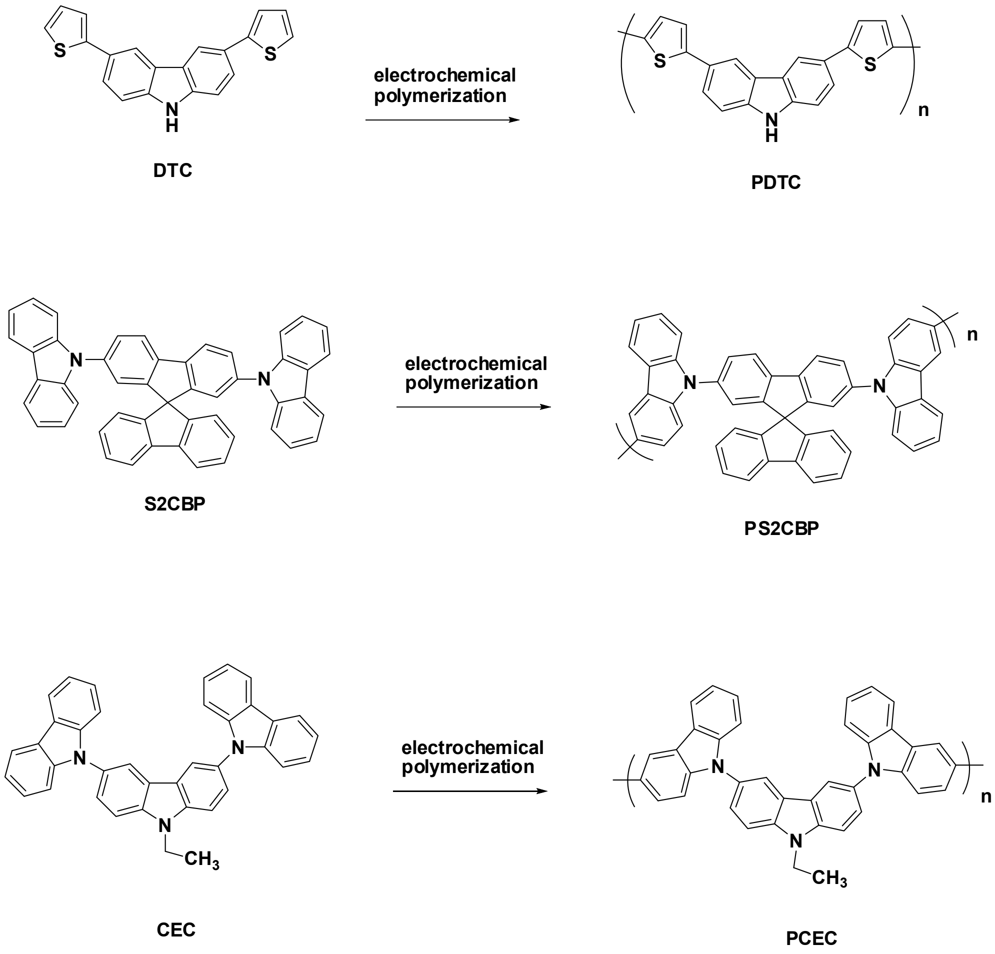

Fig. S4. The schemes for electrochemical polymerization of PDTC, PS2CBP, and PCEC.

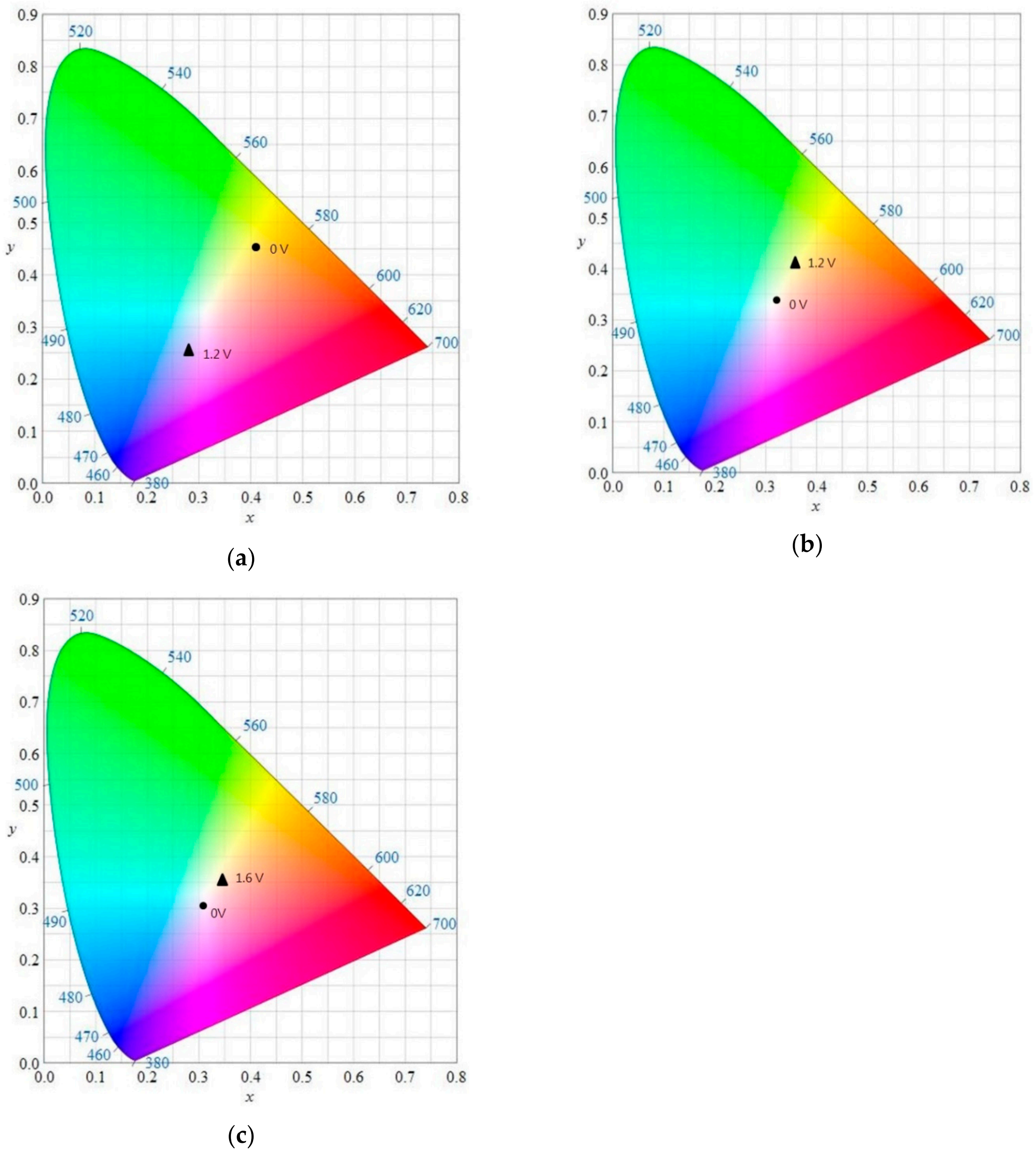

Fig. S5. CIE chromaticity diagrams of (a) PDTC film in [EPI^+][$TFSI^-$] solution at 0 V (●) and 1.2 V (▲), (b) PS2CBP film in [EPI^+][$TFSI^-$] solution at 0 V (●) and 1.2 V (▲), and (c) PCEC film in [EPI^+][$TFSI^-$] solution at 0 V (●) and 1.6 V (▲).

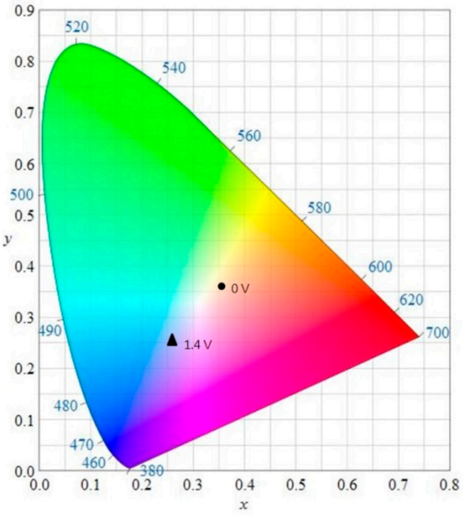

(**a**)

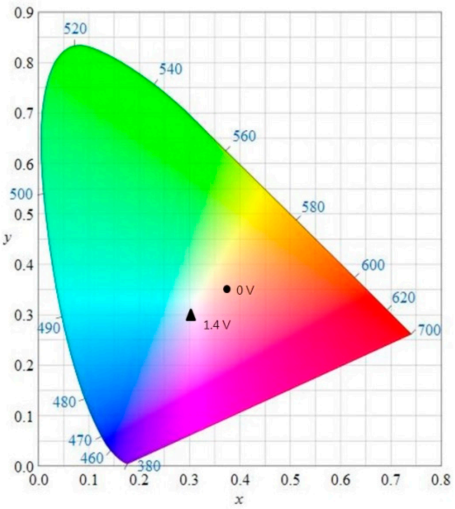

(**b**)

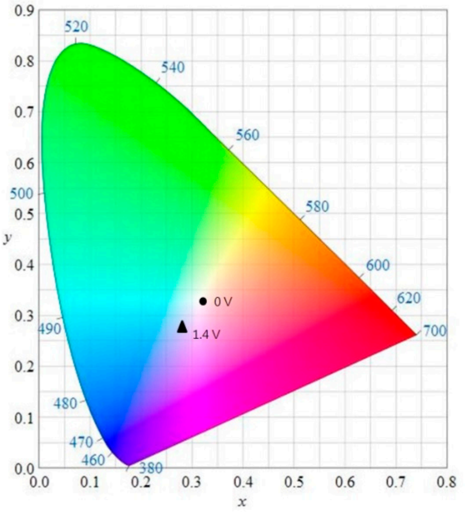

(**c**)

Fig. S6. CIE chromaticity diagrams of (a) PDTC/PProDOT-Et_2, (b) PS2CBP/PProDOT-Et_2, and (c) PCEC/PProDOT-Et_2 ECDs at 0 V (●) and 1.4 V (▲).

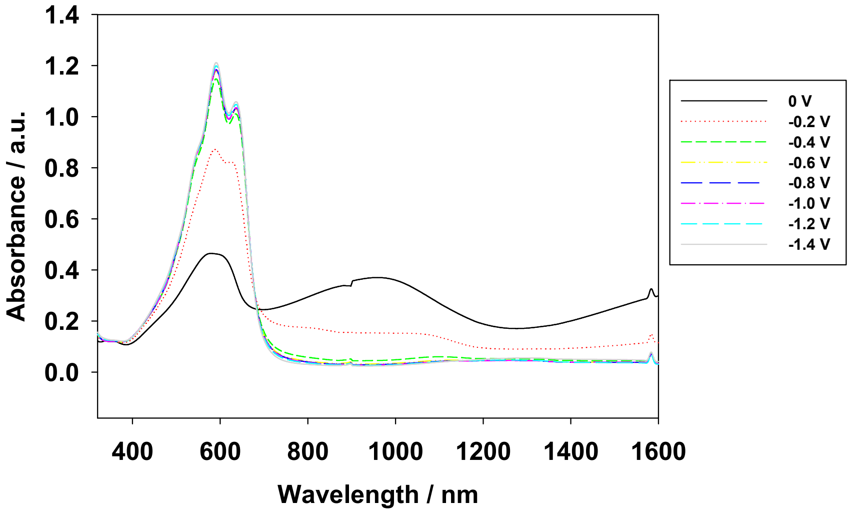

Fig. S7. Spectroelectrochemical spectra of PProDOT-Et2 film on ITO electrode at different applied potentials in $[EPI^+][TFSI^-]$ solution.

Table S1. The CIE chromaticity values (*x*, *y*) of PDTC, PS2CBP, and PCEC films at different applied potentials in $[EPI^+][TFSI^-]$.

Polymers	*x*	*y*
PDTC	0.425	0.4648
	0.333	0.3492
	0.2831	0.2631
	0.2823	0.2695
	0.2857	0.2846
PS2CBP	0.3251	0.3335
	0.3396	0.3514
	0.3729	0.402
	0.3584	0.4024
	0.3405	0.3531
	0.3479	0.3552
PCEC	0.3199	0.3264
	0.3314	0.3477
	0.338	0.3677
	0.3133	0.349
	0.3147	0.3439
	0.3302	0.3512

Table S2. The CIE chromaticity values (*x*, *y*) of PDTC/PProDOT-Et$_2$, S2CBP/PProDOT-Et$_2$, and PCEC/PProDOT-Et$_2$ ECDs at different applied potentials.

ECDs	*x*	*y*
PDTC/PProDOT-Et$_2$	0.3497	0.3577
	0.2913	0.285
	0.2769	0.2684
	0.2724	0.26
	0.2709	0.2561
PS2CBP/PProDOT-Et$_2$	0.3701	0.3564
	0.3136	0.3198
	0.3092	0.3153
	0.3066	0.3127
	0.3044	0.3106
PCEC/PProDOT-Et$_2$	0.3215	0.3345
	0.3114	0.3207
	0.2882	0.3039
	0.278	0.2974
	0.2771	0.2994
